# Supplementary material for: EXACT: a collaboration toolset for algorithm-aided annotation of images with annotation version control
Source: Sci Rep. 2021 Feb 23;11:4343. doi: 10.1038/s41598-021-83827-4 (PMC7902667; doi:10.1038/s41598-021-83827-4)
Supplement: Supplementary file 1 — Supplementary Information. [file 41598_2021_83827_MOESM1_ESM.pdf]

EXACT: A collaboration toolset for algorithm-aided annotation of images with annotation version control

Christian Marzahl1,2,*, Marc Aubreville1,4, Christof A. Bertram3, Jennifer Maier1, ChristianBergler1, Christine Kröger2, Jörn Voigt2, Katharina Breininger1, Robert Klopfleisch3, and Andreas Maier1

File	Title	Supplementary File	Caption
Annotation Templates.mp4	Annotation Templates	3	How to create annotation templates with EXACT
Segmentation.mp4	Segmentation	11	How to segment with EXACT
Density Maps.mp4	Density Maps	6	How to create density maps and explanation of the EIPH plugin
SlideRunner.mp4	SlideRunner	12	How to synchronise between SlideRunner and EXACT
ImageSets creation.mp4	ImageSets creation	7	How to create image sets and upload images
Annotation Maps.mp4	Annotation Maps	2	How to create annotation maps
Demo Dataset.mp4	Demo Dataset	5	How to setup and use a demo data set
Sound Clustering.mp4	Sound Clustering	13	How to perform sound clustering and visualisation
User Rights.mp4	User Rights	14	How to setup user access rights
ImageSets details.mp4	ImageSets details	8	Explain image set details
Versioning.mp4	Versioning	15	How to create a new image set version and track changes
Annotation download.mp4	Annotation download	1	How to download annotations Explanation of parts of the annotation study
Inference.mp4	Inference	9	Example for REST-API and JavaScript inference
Installation.mp4	Installation	10	EXACT installation guide with Docker
Asthma Clustering.mp4	Asthma Clustering	4	How to cluster Asthma cells
